# Insight into the use of tympanic temperature during target temperature management in emergency and critical care: a scoping review

**DOI:** 10.1186/s40560-021-00558-4

**Published:** 2021-06-12

**Authors:** Michela Masè, Alessandro Micarelli, Marika Falla, Ivo B. Regli, Giacomo Strapazzon

**Affiliations:** 1 Institute of Mountain Emergency Medicine, Eurac Research, Drususallee/Viale Druso 1, I-39100 Bolzano, Italy; 2 IRCS-HTA, Bruno Kessler Foundation, Trento, Italy; 3 ITER Center for Balance and Rehabilitation Research (ICBRR), Rome, Italy; 4 Centre for Mind/Brain Sciences, CIMeC, University of Trento, Rovereto, Italy; 5 Department of Anesthesia and Intensive Care, “F. Tappeiner” Hospital, Merano, Italy

**Keywords:** Target temperature management, Hypothermia, Ear canal, Tympanic membrane, Cooling devices, Hearables, Cardiac arrest, Stroke, Physiological monitoring, Temperature

## Abstract

**Background:**

Target temperature management (TTM) is suggested to reduce brain damage in the presence of global or local ischemia. Prompt TTM application may help to improve outcomes, but it is often hindered by technical problems, mainly related to the portability of cooling devices and temperature monitoring systems. Tympanic temperature (T_Ty_) measurement may represent a practical, non-invasive approach for core temperature monitoring in emergency settings, but its accuracy under different TTM protocols is poorly characterized. The present scoping review aimed to collect the available evidence about T_Ty_ monitoring in TTM to describe the technique diffusion in various TTM contexts and its accuracy in comparison with other body sites under different cooling protocols and clinical conditions.

**Methods:**

The scoping review was conducted following the guidelines of the Preferred Reporting Items for Systematic Review and Meta-Analysis extension for scoping reviews (PRISMA-ScR). PubMed, Scopus, and Web of Science electronic databases were systematically searched to identify studies conducted in the last 20 years, where T_Ty_ was measured in TTM context with specific focus on pre-hospital or in-hospital emergency settings.

**Results:**

The systematic search identified 35 studies, 12 performing T_Ty_ measurements during TTM in healthy subjects, 17 in patients with acute cardiovascular events, and 6 in patients with acute neurological diseases. The studies showed that T_Ty_ was able to track temperature changes induced by either local or whole-body cooling approaches in both pre-hospital and in-hospital settings. Direct comparisons to other core temperature measurements from other body sites were available in 22 studies, which showed a faster and larger change of T_Ty_ upon TTM compared to other core temperature measurements. Direct brain temperature measurements were available only in 3 studies and showed a good correlation between T_Ty_ and brain temperature, although T_Ty_ displayed a tendency to overestimate cooling effects compared to brain temperature.

**Conclusions:**

T_Ty_ was capable to track temperature changes under a variety of TTM protocols and clinical conditions in both pre-hospital and in-hospital settings. Due to the heterogeneity and paucity of comparative temperature data, future studies are needed to fully elucidate the advantages of T_Ty_ in emergency settings and its capability to track brain temperature.

## Background

Targeted temperature management (TTM), former therapeutic hypothermia, is an intentional reduction of core temperature to a selected and strictly controlled [1] range of values, which is aimed to improve outcomes in various clinical conditions, including cardiac arrest (CA), traumatic brain injury, stroke, and myocardial infarction [2–4]. By lowering brain temperature, TTM is thought to mitigate brain damage due to global (i.e., CA) or local (i.e., stroke) ischemia, through various mechanisms, including a decrease of cerebral oxygen and glucose consumption, and a reduction of ATP demand [5, 6]. Current guidelines and recent trials support the use of TTM (in the range of 32–36 °C [2, 3]) in all CA patients who remain in a state of coma after return of spontaneous circulation (ROSC) [2, 7–11]. The benefit of systemic and selective TTM in stroke patients is supported by recent trials and meta-analyses [12, 13]. Despite a broad consensus on TTM benefits, the application of pre-hospital TTM, for example in out-of-hospital CA, is still controversial [14, 15]. Variable outcomes have been reported [16–18], which may be partially due to limitations in pre-hospital cooling procedures and/or accuracy of temperature monitoring.

Since discrepancies between brain and systemic temperatures have been described, direct monitoring of brain temperature would be desirable for optimal TTM [19]. However, brain temperature measurement techniques are invasive and impractical in most cases and settings. Among different sites for core temperature measurement (e.g., ear canal, rectum, bladder, esophagus, and pulmonary artery) [20], the ear canal (or tympanic membrane) has been proposed as a surrogate measurement site during TTM procedures, especially in pre-hospital and emergency settings, thanks to its accessibility, minimal invasiveness, and fast response. The vasculature pattern of the tympanic membrane is shared with the brain and mediates a thermal equilibrium between the two sites [21–23], which suggests the potential of tympanic temperature (T_Ty_) to reflect brain temperature. In addition, the vasculature in the tympanic region is minimally influenced by the thermoregulatory vasomotor response, which guarantees adequate flow conditions [24]. In pre-hospital settings, T_Ty_ has been shown—albeit with mixed results—to be comparable to invasive temperature measurements at hospital admission [20], providing that insulation from the environment is ensured during measurement [25]. On the other hand, T_Ty_ measurement can only be performed if the ear canal is not obstructed (e.g., by blood, cerumen, snow) [20]. T_Ty_ can be biased in situations during which blood flow is absent or inadequate [23, 26], and/or it can be affected by anatomical and vascular changes following major ear surgery and large tympanic membrane perforations [27]. T_Ty_ accuracy under different TTM protocols (e.g., local versus whole body), TTM phases (e.g., induction versus maintenance), and different pathophysiological conditions need to be further clarified.

This scoping review aims to identify and to summarize all the available evidence over the last 20 years about T_Ty_ monitoring in the context of TTM from studies performed either in patients with various acute disorders or in healthy subjects. We describe the level of diffusion of the techniques in various TTM contexts with a focus on pre-hospital and in-hospital emergency settings. We provide indications on the accuracy of tympanic measurements in comparison to other body sites under different TTM phases, cooling protocols, and clinical conditions.

## Methods

The scoping review was conducted following the guidelines of the Preferred Reporting Items for Systematic Review and Meta-Analysis (PRISMA) extension for scoping reviews (PRISMA-ScR) [28].

### Eligibility criteria

The literature search was performed by two authors (AM and MM) to identify studies, conducted in the last 20 years, that used T_Ty_ during TTM approaches. The search strategy is schematized by the inclusion criteria in Table 1, categorized according to the broad Population-Concept-Context (PCC) mnemonic, recommended for scoping reviews [29, 30]. The scoping review was focused on pre-hospital and in-hospital emergency settings. We considered both studies testing TTM approaches in healthy subjects and studies where TTM was performed in patients experiencing different emergency conditions. Studies about accidental hypothermia, drug-induced hypothermia, and perioperative and postoperative hypothermia were excluded. The range of TTM temperatures was set to 32–36 °C according to TTM definition in [2, 3], while studies on normothermia maintenance in patients with fever were not considered. The search was restricted to articles published in English in peer-reviewed journals. No restriction on study design was posed. Abstracts presentations, conference proceedings, and reviews were excluded.

**Table 1:** Inclusion criteria for the scoping review summarized according to the Population-Concept-Context (PCC) mnemonic, recommended for scoping reviews [29, 30]

**Population**	• Healthy adults (testing of target temperature management approaches). • Patients undergoing target temperature approaches under emergency conditions, including cardiovascular and neurologic emergencies. • Any gender.
**Concept**	• Tympanic temperature measurement in the context of target temperature management.
**Context**	• Testing of target temperature management approaches in healthy subjects; target temperature management in patients in pre-hospital and in-hospital emergency settings. • Original peer-reviewed research articles (any study design), published in English in the last 20 years.

### Information sources, search strategy, and study selection

A systematic search was performed in PubMed, Scopus, and ISI Web of Science electronic databases to identify primary references from January 2000 to April 2020. The following search strings were used: (“aural” OR “tympanic” OR “epitympanic” OR “ear” OR “ear canal” OR “in-ear” OR “ear-in” OR “earbud” OR “earpiece” OR “earable”) AND (“temperature” OR “temperature monitoring” OR “core temperature” OR “core body temperature” OR “body temperature”) AND (“hypothermia” OR “hypothermic” OR “therapeutic hypothermia” OR “hypothermic treatment” OR “target temperature management” OR “TTM” OR “body cooling” OR “low temperature” OR “low body temperature”). The database search was followed by a review of the citations from eligible studies. Studies were selected based on title and abstract using the online platform Rayyan [31]. Selected studies were read thoroughly to identify those suitable for inclusion in the scoping review.

### Data extraction

Two reviewers (MM and AM) independently extracted the demographic and experimental data from the selected studies. When disagreement occurred, they reviewed the papers together to reach consensus. For each study, the following relevant information was extracted and summarized: the characteristics of the investigated study population; TTM protocols (body cooling modality, target temperature); the experimental and/or clinical settings of application; the available temperature measurements (presence and location of comparative/reference temperature measurements in addition to the tympanic one); and the main results of the studies in terms of feasibility of the tympanic measurements and comparability of T_Ty_ with core temperature measurements from other body sites.

## Results

### Selected studies

**Fig. 1:**
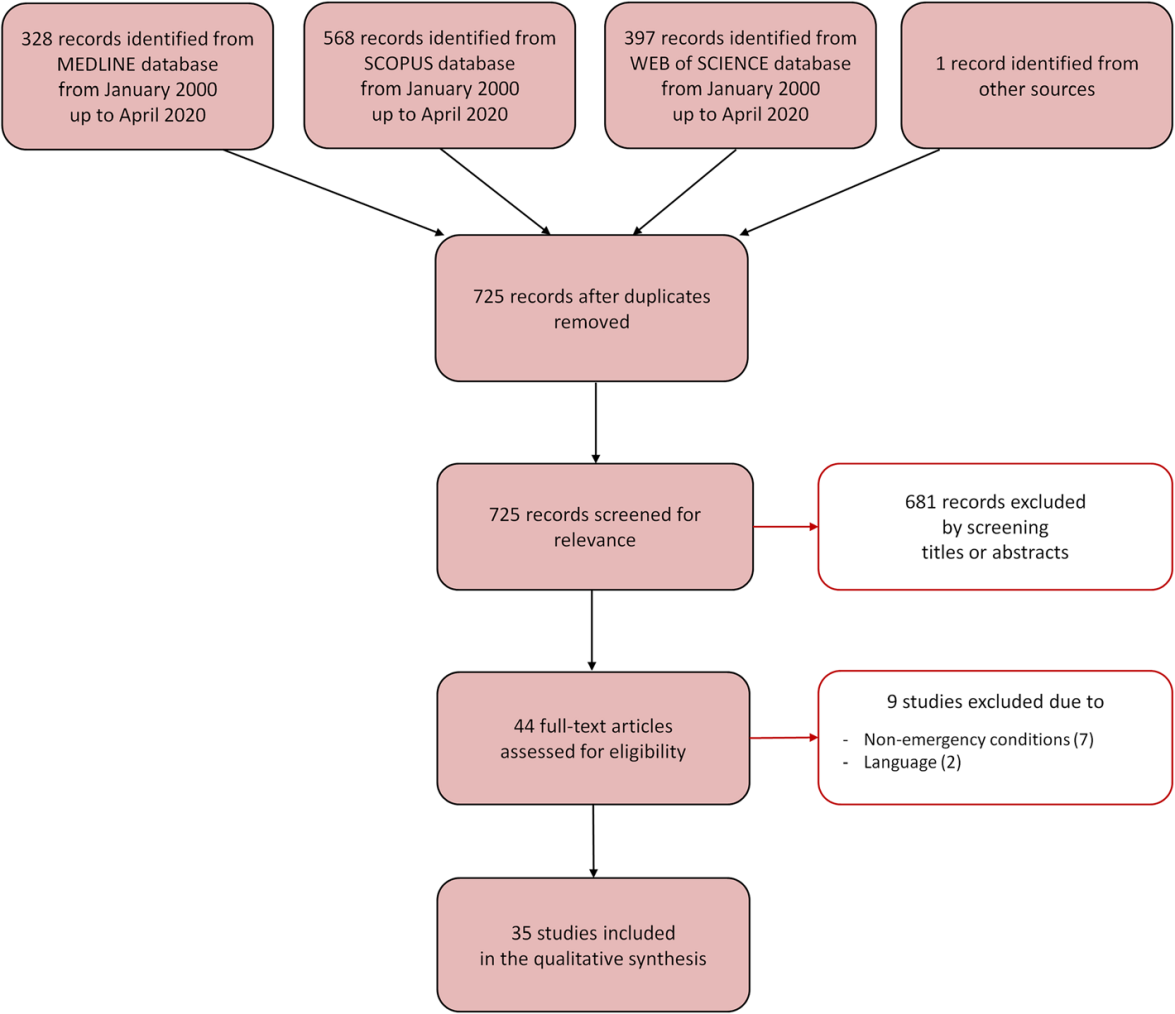
Selection process for the studies included in the scoping review. The Preferred Reporting Items for Systemic Reviews and Meta-Analyses (PRISMA-ScR) flow diagram depicts the number of records identified, included, and excluded, and the reasons for exclusion, through the different phases of the scoping review

The database search identified a total of 725 relevant references once duplicates were removed (Fig. 1). A total of 681 references were excluded after reading title and abstract and 44 were retrieved for further evaluation. Of these, 9 studies were excluded, because they did not fulfill the inclusion criteria. Following the selection process, 35 studies were included in the scoping review. Of these studies, 12 measured T_Ty_ during tests of TTM protocols in healthy subjects, 17 during TTM in patients with acute cardiovascular events, and 6 during TTM in patients with acute neurological disorders. The studies are described in the next paragraphs and summarized in Tables 2, 3, and 4.

**Table 2:** Studies testing different approaches of target temperature management in healthy subjects.

Study	Population	Cooling approach		Tympanic TM device	Core TM sites	Other TM sites	Main results	
		Location	Protocol				Feasibility	Comparability
**Bagi**ć **et al.** [32]	N: 10 Age: [21–47] y Male: 50%	Head and neck cooling	Cooling session lengths: 30 and 60 min. Cooling device T: 1.5 to 4.5–5 °C.	IRTT, Braun PRO 3000 Thermometer, Braun GmbH, Germany; T_Ty_ measured in both ears.	Intestinal pill	Scalp, forearm, abdomen, leg, face, and mouth	In both ears, T_Ty_ displayed significant differences between the start and end of cooling (p < 0.001).	A significant difference was observed in scalp T (p < 0.001), but not in intestinal T (p = NS).
**Kallmünzer et al.** [33]	N: 10 Age: 35 (28–42) y Male: 60%	Neck cooling	Cooling session length: 190 min Cooling device T: 4 °C.	IRTT, Genius 2, Tyco Healthcare Group, USA	Rectal	None	T_Ty_ displayed a significant drop after neck cooling (−1.7 °C, p = 0.001).	Rectal T displayed a smaller decrease (−0.65 °C, p = 0.019).
**Koehn et al.** [34]	N: 11 Age: 42 ± 11 y Male: 45%	Neck cooling	Cooling session length: 90 min. Cooling device T: 4 °C.	Thermocouple thermometer, ELan Med GmbH, Germany	Rectal	Neck skin	T_Ty_ showed a slight but significant decrease (from 35.6 ± 0.2 °C to 35.0 ± 0.8 °C, p = 0.026) within 10 min of cooling, reaching minimal values (34.7 ± 0.4 °C, p < 0.001) after 50 min	Neck skin and rectal T decreased respectively by a higher and lower extent than T_Ty_.
**Koehn et al.** [35]	N: 10 Age: 35 ± 13 y Man: 100%	Head and neck cooling	Cooling session length: 120 min Cooling device T: 4 °C.	Thermocouple thermometer, ELan Med GmbH, Germany	Rectal	Forehead skin	T_Ty_ decreased to minimal values (from 36.6 ± 0.7 °C to 31.8 ± 1.2 °C, p < 0.001) after 40 min of cooling, with a slow increase thereafter.	Forehead skin and rectal T achieved the respective lowest values at 20 and 120 min, respectively.
**Zweifler et al.** [36]	N: 22 Age: 31 ± 8 y Male: 45%	Chest and thighs cooling	Active cooling plus hypothermia maintenance: < 5 h. TT: 34–35 °C (tympanic). Shivering suppression by meperidine, buspirone, and MgSO_4_.	Thermocouple thermometer, Mon-a-Therm, Mallinckrodt Anesthesia Products, USA; ear canal occluded with cotton and gauze, ear probe taped in place.	Rectal	None	T_Ty_ reached the TT=35 °C in a median time of 88 min (mean cooling rate of 1.4 ± 0.5 °C/h).	A time-dependent gradient was observed between T_Ty_ and rectal T (from −0.1 ± 0.3 °C at baseline to −0.6 ± 0.4 °C at 105 min, −0.3 ± 0.5 °C at maintenance phase).
**Zweifler et al.** [37]	Intervention 1: N: 8 Age: 33 ± 8 y Male: 37% Intervention 2: N: 14 Age: 30 ± 9 y Male: 36%	Chest and thighs cooling	Active cooling plus hypothermia maintenance: < 5 h. TT: 34–35 °C (tympanic). Shivering suppression by meperidine, buspirone, or ondansetron, with (intervention 1) or without MgSO_4_ (intervention 2).	Thermocouple thermometer, Mon-a-Therm, Mallinckrodt Anesthesia Products, USA; ear canal occluded with cotton and gauze, ear probe taped in place.	Rectal	None	Baseline T_Ty_ was 36.8±0.2 °C in intervention 1 and 37.0±0.3 °C in intervention 2. T_Ty_ depicted the prolongation of cooling time induced by meperidine (delay of 36 min, p = 0.003, for each 50 mg of drug) and the reduction of cooling time by MgSO_4_ (17 min, p = 0.039).	Baseline rectal T was 37.0±0.2 °C in intervention 1 and 37.0±0.3 °C in intervention 2.
**Zweifler et al.** [38]	Intervention 1: N: 5 Age: 36 ± 5 y Male: 60% Intervention 2: N: 5 Age: 30 ± 11 y Male: 20%	Intervention 1: Chest and thighs cooling Intervention 2: Thighs, back, and abdomen cooling	Intervention 1: Active cooling plus hypothermia maintenance: < 5 h. TT: 34–35 °C (tympanic). Intervention 2: Active cooling plus hypothermia maintenance: < 5 h. TT: 34.5 °C (rectal). In both, shivering suppression by meperidine, or chlorpromazine	Thermocouple thermometer, Mon-a-Therm, Mallinckrodt Anesthesia Products, Inc, USA.	Rectal	Mean skin-surface T from calf, thigh, chest, and upper arm skin.	T_Ty_ reached the TT=35 °C in 77 ± 23 min (mean cooling rate of 1.5 ± 0.6 °C/h) in Intervention 1 and in 90 ± 53 min (mean cooling rate of 1.4 ± 0.4 °C/h) in Intervention 2.	Rectal T displayed higher values than T_Ty_ over the cooling procedure in intervention 2.
**Mahmood et al.** [39]	N: 18 Age: 32 ± 8 y Male: 44%	Chest and thighs cooling	Active cooling plus hypothermia maintenance: ≤ 5 h. TT: 34.5 °C (tympanic). Shivering suppression by meperidine and/or ondansetron and/or buspirone.	NR	Rectal	None	T_Ty_ changes correlated with the mean flow velocity of the middle cerebral artery (p < 0.001).	At baseline T_Ty_ was 36.9 ± 0.3 °C, while rectal T was 37.0 ± 0.2 °C.
**Adams and Koster** [40]	N: 10 Age: > 18 y Male: 50%	Face and neck cooling	Device application at ambient T of 19 °C.	IRTT, Genius 3000A, Sherwood-Davis & Geck, Gosport UK	None	None	T_Ty_ showed a difference of 0.433 °C (p < 0.0001) between baseline and end of cooling exposure.	NR
**Doufas et al.** [41]	N: 10 Age: 24 ± 4 y Male: 100%	Whole body cooling by lactated Ringer’s solution (~4 °C)	Lactate infusion to decrease T_Ty_ by 1–2 °C/h until identification of the shivering threshold. Conditions tested: no drug, dexmedetomidine and/or meperidine.	Thermocouple thermometer, Mon-a-Therm, Mallinckrodt Anesthesiology Products, Inc., Ireland; ear canal occluded with cotton, probe taped in place, bandage over the ear.	None	Mean skin surface T from 15 area-weighted sites.	T_Ty_ detected the significant (p < 0.001) reduction of the shivering threshold induced by meperidine (drop of 1.2°C), dexmedetomidine (0.7 ± 0.5 °C), and their combination (2.0 ± 0.5 °C).	NR
**Jackson et al.** [42]	N: 12 Age: 27 ± 11 y Male: 42%	Head and neck cooling	Cooling session length: 90 min. Cooling device settings: (i) maximum cooling; (ii) bypass mode in each participant.	IRTT, Genius 2, Tyco Healthcare Group, USA	None	Sublingual	In condition (i), T_Ty_ decreased from 37.01 ± 0.34 °C to 36.70 ± 0.38 °C (60 min) and to 36.76 ± 0.33 °C (90 min). In (ii), T_Ty_ decreased to a smaller extent, from 36.93 ± 0.30 °C to 36.85 ± 0.29 °C (60 min) and 36.85 ± 0.27 °C (90 min).	Sublingual T showed a slower response. In (i), it decreased from 36.80 ± 0.14 °C to 36.70 ± 0.10 °C (60 min) and to 36.70 ± 0.12 °C (90 min). In (ii), it decreased from 36.74 ± 0.12 °C to 36.72 ± 0.11 °C (60 min) and 36.71 ± 0.08 °C (90 min).
**Wadhwa et al.** [43]	N: 9 Age: 27 [18–40] y Male: 100%	Whole body cooling by lactated Ringer’s solution (~4 °C)	Lactate infusion via central venous catheter for 2 h to decrease T_Ty_ by ≈1.5 °C·h^−1^. Condition tested: intravenous MgSO_4_ (bolus of 80 mg·kg^−1^ plus infusion of 2 g·h^−1^), or an equal volume of saline solution.	Thermocouple thermometer, Tyco-Mallinckrodt Anesthesiology Products, Inc, USA; ear canal occluded by cotton and gauze.	None	Skin surface	T_Ty_ detected a significant reduction of the shivering threshold (0.3 ± 0.4 °C, p = 0.040) by MgSO_4_ infusion. T_Ty_ was 36.6 ± 0.2 °C after 30 min of MgSO_4_ infusion vs. 36.8 ± 0.3 °C after 30 min of saline solution infusion.	Skin T was 33.2 ± 0.7 °C after 30 min of MgSO_4_ infusion vs. 33.6 ±1.3 °C after 30 min of saline solution infusion.

Data are numbers (N), percentages (%), mean ± standard deviation, or [range], as available. *HR*, heart rate; *IRTT*, infrared tympanic thermometer; *MgSO*_*4*_, magnesium sulfate; *NR*, not reported; *T*_*Ty*_, tympanic temperature; *T*, temperature; *TM*, temperature measurement: *TT*, target temperature; *vs*., versus; *y*, years

**Table 3:** Studies performing target temperature management in acute cardiovascular events

Study	Pathology	Population	Cooling approach	Setting	Tympanic TM device	Core TM sites	Other TM sites	Main results	
								Feasibility	Comparability
**Busch et al.** [44]	CA	N: 84 Age: 71 (63; 79) y Male: 76%	Post-ROSC trans-nasal cooling. TT: 33 °C (tympanic and esophageal).	ED/ ICU	IRTT, ThermoScan Pro 4000, Braun GmbH, Germany	Esophageal, or arterial, or bladder, or rectal	None	T_Ty_ displayed a cooling rate of 2.3 (1.6; 3.0) °C/h. The cooling time to reach the tympanic TT was 60 (36.5; 117.5) min and was reached in 66% of pts.	The cooling rate of overall core sites (esophageal, arterial, bladder, or rectal) was 1.1 (0.7; 1.5) °C/h, with a faster response for esophageal or arterial (1.4 (0.9; 2.0) °C/h) than for bladder or rectal (0.9 (0.5; 1.2) °C/h; p=0.001). The cooling time to reach the core TT was 180 (120; 285) min and was reached in 19% of patients.
**Callaway et al.** [45]	OHCA	Intervention: N: 9 Age: 68 ± 15 y Male: 100% Control: N: 13 Age: 80 ± 10 y Male: 71%	Intervention: intra-arrest head and neck cooling. TT: 34 °C (esophageal). Control: standard care.	PH/ ED	IRTT, NR.	Esophageal	Naso-pharyngeal	T_Ty_ displayed unpredictable variations due to ice in the ears.	NR
**Castren et al.** [46]	OHCA	Intervention: N: 93 Age: 66 y Male: 72% Control: N: 101 Age: 64 y Male: 78%	Intervention: intra-arrest trans-nasal cooling. TT: 34 °C (tympanic and core). Control: standard care.	PH	IRTT, NR	Rectal, or bladder, or intravascular	None	T_Ty_ at hospital arrival was significantly different (p<0.001) in intervention (34.2±1.5 °C) vs. control (35.5±0.9 °C). The cooling rate was 1.3 °C in 26 min. The cooling time to reach the tympanic TT was significantly shorter (p=0.03) in the intervention (102 (81; 155) min) vs. the control (291(183; 416) min) group.	Core T (rectal, or bladder, or intravascular) at admission was 35.1±1.3 °C in intervention vs. 35.8 °C ± 0.9 °C in control (p<0.01). Cooling time to reach the core TT was 155 (124; 315) min in the intervention vs. 284 (172; 471) in the control group.
**Hasper et al.** [47]	OHCA	N: 10 Age: 71.5 y^§^ Male: 80%	Post-ROSC whole body cooling by cold saline infusion and water pads. TT: 33 °C (NR).	ED	IRTT, Braun ThermoScan pro4000; Welch Allyn, Germany	Esophageal, or bladder	None	During TTM T_Ty_ was 33.40 {33.30; 33.60} °C.	T_Ty_ displayed a small bias with respect to esophageal T (0.021 °C ± 0.80 °C) and a high significant correlation with esophageal (r=0.95, p<0.0001) and bladder T (r=0.96, p<0.0001).
**Hachimi-Idrissi et al.** [48]	OHCA	Intervention: N: 16 Age: 77 [52; 95] y Male: 56% Control: N: 14 Age: 74 [59; 91] y Male: 64%	Intervention: head and neck cooling. TT: 34 °C (bladder). Control: standard care.	ED	IRTT, Braun Thermoscan, Braun, Germany	Central venous, arterial, or bladder	Scalp	The cooling time to reach the tympanic TT in intervention was 60 (15; 240) min.	The cooling time to reach the bladder TT was longer, 180 (70; 240) min (p = NR).
**Islam et al.** [49]	OHCA	Intervention: N: 37 Age: 64 ± 12 y Male: 86% Control: N: 37 Age: 62 ± 13 y Male: 74%	Intervention: post-ROSC intra-nasal cooling. TT: 34 °C (tympanic and esophageal). Control: standard surface-cooling. TT: 34 °C (tympanic and esophageal).	CL by direct admission	NR	Esophageal	None	In the first cooling hour, T_Ty_ showed a significantly larger drop (1.75 °C) in intervention vs. control (0.935 °C, p<0.01). The cooling time to reach the tympanic TT was 75.2 min in intervention vs. 107.2 min in control (p=NS).	Esophageal T drop in the first hour was not significantly different in intervention (1.148 °C) vs. control (0.904 °C, p = NS). The cooling time to reach the esophageal TT was 84.7 min in intervention vs. 114.9 min in control (p=NS).
**Krizanac et al.** [50]	OHCA	N: 20 Age: 63 (43; 88) y Male: 80%	Post-ROSC cooling by cooling pads, or intravascular cooling catheters and intravenous cold saline infusion. TT: 33 °C (esophageal).	ED	Thermistor thermometer, Mon-a-Therm, Tyco Healthcare, UK.	Esophageal, bladder, pulmonary artery, or femoral-iliac artery	None	T_Ty_ tracked temperature changes induced by cooling but continuously and substantially underestimated the pulmonary artery T during cooling as well as during steady state.	The bias of T_Ty_ compared to pulmonary artery T were -0.6 {−0.8; −0.3} °C (overall) and −0.6 {−0.8; −0.4}°C (cooling phase). The tympanic TT was reached with an anticipation of −38 {−65; −23.5} min compared to the pulmonary artery.
**Shin et al.** [51]	OHCA	N: 21 Age: 50 ± 20 y Male: 71%	Post-ROSC cooling by cold saline infusion and external cooling pads. TT: 33 °C (bladder).	ED	Thermistor thermometer, Probe 400 Series, DeRoyal, USA; inserted after otoscopic exam, taped in place, covered with bandage.	Rectal, bladder, or pulmonary artery	None	T_Ty_ tracked the changes induced by cold saline cooling, but it underestimated pulmonary artery T through the whole procedure.	The bias^$^ of T_Ty_ compared to pulmonary artery was: −1.03 ± 1.47 °C (overall), −1.11 ± 1.53 °C (induction phase), −1.12 ± 1.29 °C (maintenance phase), and −0.89 ± 1.62 °C (rewarming phase). The correlation was: 0.860 (overall), 0.815 (induction phase), 0.611 (maintenance phase), and 0.776 (rewarming phase).
**Stratil et al.** [52]	CA	Winter group (outside T ≤ 10 °C): N: 61 Age: 60 (50; 75) y Male: 70% Summer group: (outside T ≥ 20 °C): N: 39 Age: 57 (48; 65) y Male: 77%	Mild therapeutic hypothermia by surface or invasive cooling in 25 winter and 24 summer patients. TT: <34 °C (NR).	ED	IRTT, Ototemp LighTouch; Exergen, USA; only at admission.	Bladder, or esophageal	None	T_Ty_ at hospital admission was significantly lower (p=0.001) in winter (34.9 °C (34; 35.6)) vs. summer group (36 °C (35.3–36.3)).	Core T at admission was 35.3 °C (34.8; 35.9) in winter vs. 36.2 °C (35.5–36.7) in summer group (p = 0.001).
**Takeda et al.** [53]	CA (mainly OHCA)	Intervention: N: 53 Age: 72 (62; 81) y Male: 47% Control: N: 55 Age: 72 (64; 78) y Male: 67%	Intervention: pre- or post-ROSC pharyngeal cooling plus whole body cooling. TT: 32 °C (tympanic). Control: standard care.	ED	Thermistor thermometer, TM400, Covidien, Japan; T_Ty_ measured bilaterally, insulation with adhesive wrapping material.	Rectal, or bladder	None	In intervention T_Ty_ showed a drop of 0.06 °C/min in the first 10 min after arrival, followed by a slower decrease. T_Ty_ was significantly lower in intervention vs. control at 40 min (33.7 ± 1.4 °C vs. 34.1 ± 1.1 °C, p = 0.02) and 120 min (32.9 ± 1.2 °C vs. 34.1 ± 1.3 °C, p < 0.001).	Core T dropped by 0.02 °C/min at 30 min after arrival. Core T was significantly lower in intervention vs. control at 120 min (34.5 ± 1.1 °C vs. 35.3 ± 1.0 °C, p = 0.02).
**Wandaller et al.** [54]	CA	Intervention 1: N: 5 Intervention 2: N: 6 Control: none. Demographic data: NR.	Intervention: post-ROSC head cooling without (1) or with neck cooling (2). Additional endovascular cooling if necessary. TT: 33 °C (esophageal).	ED	Thermocouple thermometer, Mon-a-term, Mallinckrodt, Inc, USA.	Esophageal and jugular	None	T_Ty_ showed a drop of 3.4 °C in the first 3 h of cooling.	T drop was 3.7 °C at the jugular site and 2.4 °C at the esophageal site. With respect to esophageal T, T_Ty_ displayed a bias of − 1.65 {−2.2; −1.1} °C (p=0.001) in Intervention 1 vs. −3.06 {-4.27; −1.85} °C in Intervention 2 (p=0.001).
**Zeiner et al.** [55]	OHCA	N: 27 Age: 58 (52; 64) y Male: 74%	Post-ROSC surface body cooling plus head and body cooling by pre-cooled mattress. TT: 33 ± 1 °C (pulmonary artery).	ED/ICU	IRTT, Ototemp LighTouch, Exergen, USA; only at admission.	Esophageal, bladder, or pulmonary artery	None	T_Ty_ was measured only at admission and showed a value of 35.3 °C (34.9–36.0 °C).	NR
**Ko et al.** [56]	OHCA	Intervention: N: 23 Age: 55 ± 15 y Male: 87% Control: N: 35 Age: 63 ± 18 y Male: 71%	Intervention: post-ROSC whole body cooling by blanket and cold crystalloid intravenous infusion. TT: 33 °C (tympanic). Control: standard care.	ED/ ICU	Non-contact thermometer, NR.	None	None	T_Ty_ detected significant differences (p = 0.004) during TTM in intervention (35.16 °C) vs. control (36.5 °C).	NR
**Skulec et al.** [57]	OHCA	Intervention: N: 40 Age: 61 ± 18 y Male: 85% Control: N: 40 Age: 61 ± 17 y Male: 72%	Intervention: post-ROSC, PH cooling by intravenous cold saline infusion plus in-hospital TTM. TT: <34 °C (tympanic). Control: standard care (in-hospital TTM).	PH/ ED	NR	None	None	In intervention, T_Ty_ dropped by 1.4 ± 0.8 °C (from 36.2 ± 1.5 to 34.7 ± 1.4 °C; p<0.001) in 42.8 ± 19.6 min. The tympanic TT was reached in 17.5% of patients.	NR
**Storm et al.** [58]	OHCA	Intervention: N: 20 Age: 62 (52; 71) y Male: 65% Control: N: 25 Age: 58 (44; 66) y Male: 84%	Intervention: post-ROSC head cooling by hypothermia cap. TT: NR. Control: standard care.	PH	NR	None	None	In intervention: T_Ty_ dropped from 35.5 °C (34.8; 36.3) to 34.4 °C (33.6; 35.4) after head cooling (p<0.001). In two patients, T_Ty_ was not affected by cooling.	NR
**Erlinge et al.** [59]	STEMI	Intervention: N: 61 Age: 57 (37; 79) y Male: 79% Control: N: 59 Age: 59 (30; 75) y Male: 86%	Intervention: pre-reperfusion cooling by cold saline infusion. TT: ≤35 °C (cooling catheter) before reperfusion. Control: standard care.	CL	NR	None	Cooling catheter sensor, during endovascular cooling	In the intervention group, T_Ty_ was measured only at baseline and was 36.0 ± 0.7 °C.	The cooling catheter T at reperfusion was 34.7 ± 0.6 °C (p=NR).
**Testori et al.** [60]	STEMI	Intervention: N: 47 Age: 58 ± 10 y Male: 79% Control: N: 54 Age: 55 ± 12 y Male: 81%	Intervention: PH cooling by cold saline and surface pads, followed by CL endovascular cooling. TT: 34 °C (cooling catheter). Control: standard care.	PH / CL	IRTT, Ototemp Ligh-Touch, Exergen, USA	None	Cooling catheter sensor, during endovascular cooling	In the intervention group, T_Ty_ displayed a significant decrease from a baseline of 36.1 ± 0.5 °C to 35.5 ± 0.6 °C after PH cooling (p < 0.01).	The cooling catheter T at reperfusion was 34.4 °C ± 0.6 °C.

Data are numbers (N), percentages (%), mean, mean ± standard deviation or limits of agreements*, mean {95% confidence interval}, median^§^, median (interquartile range), median [range], as available. ^$^, bias definition reversed with respect to the original publication. *CA*, cardiac arrest; *CL*, catheter lab; *ED*, emergency department; *ICU*, intensive care unit; *IRTT*, infrared tympanic thermometer; *NR*, not reported; *NS*, not significant; *OHCA*, out of hospital cardiac arrest; *MI*, myocardial infarction; *PH*, pre-hospital; *r*, correlation coefficient; *ROSC*, return of spontaneous circulation; *STEMI*, ST-elevation myocardial infarction; *T*, temperature; *TM*, temperature measurement; *TT*, target temperature; *T*_*Ty*_, tympanic temperature; *y*, years; *vs.*, versus

**Table 4:** Studies performing target temperature management in acute neurologic diseases

Study	Disease	Population	Cooling approach		Setting	Tympanic TM device	Core TM site	Other TM sites	Main results	
			Location	Protocol					Feasibility	Comparability
**Abou-Chebl et al.** [61]	TBI, IS, ICH	N: 15* Age: 50 ± 17 y Male: 40% NIHSS: 26.7 ± 6.7	Naso-pharingeal cooling	Administration rate of 80 L/min for 1 h.	NICU	NR	Bladder, or rectal, or esophageal, or pulmonary artery. Brain	None	T_Ty_ decreased by 2.2 ± 2 °C during induction, with a drop of 0.65 ± 0.39 °C within 15 min (two outliers excluded).	During induction, T decreased by 1.4 ± 0.4 °C (by 0.53 ± 0.24 °C at 15 min) at the brain and by 1.1 ± 0.6 °C (by 0.43 ± 0.35 °C at 15 min) at core sites (bladder, rectal esophageal, or pulmonary artery).
**Poli et al.** [62]	IS, ICH, SAH	Intervention 1: N: 10 Age: 65 ± 7 y Male: 60% Intervention 2: N: 10 Age: 56 ± 12 y Male: 50% Overall NIHSS: 14.5 (6.75-24.75)	Intervention 1: Whole body cooling by cold saline infusion (4 °C). Intervention 2: Naso-pharingeal cooling	Intervention 1: Infusion flow rate of 4 L/h for 33 ± 4 min. Intervention 2: rate of 60 L/min for 1 h.	NICU	NR	Bladder, rectal, and esophageal. ICP/T brain probe (>3 cm below the cortical surface)	None	T_Ty_ reacted similarly to relative changes of brain T during cold infusion, albeit with slightly different absolute values.	T_Ty_ correlated well with brain T changes induced by cold infusion, but overestimated brain cooling by naso-pharyngeal cooling (p = 0.005). T_Ty_ was slightly lower than brain T even at baseline (37.1 ± 0.7 °C vs 37.5 ± 0.7 °C, p < 0.001).
**Poli et al.** [63]	IS, ICH, SAH	N: 11 Age: 58 ± 15 y Male: 73% NIHSS: 22.9 ± 13.2	Head and neck cooling (4 °C); subsequent whole-body surface cooling if requested.	Device applied if body core T > 37.1 °C. Drugs: If body core T > 37.1 °C after 1 h of cooling or initial body core T > 38.0 °C, administration of acetaminophen or metamizole and whole-body surface cooling.	NICU	Thermistor thermometer, TTS 400, Smiths Medical, USA	Bladder. ICP/T brain probe, (>3 cm below the cortical surface)	None	After 1 h of cooling, T_Ty_ was reduced by −1.69 ± 1.19 °C (p < 0.001), with a maximum decrease of −1.79 ± 1.19 °C after 37 ± 16 min.	T_Ty_ at baseline and during cooling was significantly lower (p <0.001) than brain T. After 1 h of cooling, brain T was reduced by −0.32 ± 0.2 °C (p <0.001), and bladder T by −0.18 ± 0.15 °C (p = 0.003). The maximal decrease of brain T was −0.36 ± 0.22 °C after 49 ± 17 min, and of bladder T −0.25 ± 0.15 °C after 48 ± 19 min.
**Kammersgaard et al.** [64]	IS, ICH	Intervention: N: 17 Age: 69 ± 16 y Male: 71% SSS: 25.8 (11.5) Control: N: 56 Age: 70 ± 10 y Male: 77% SSS: 28 (11.5).	Intervention: whole-body cooling by “forced air” method. Control: standard care.	Intervention: device applied for 6 h.	SU	IRTT, Diatek Model 9000, Diatek Inc, USA	Rectal	None	The mean T_Ty_ decreased significantly after 1 h of cooling (from 36.8 °C at baseline to 36.4 °C, p = 0.002). The lowest T_Ty_ was achieved after 6 h (35.5 °C, p = 0.001 vs baseline).	A strong correlation was observed between rectal T and T_Ty_.
**Kollmar et al.** [65]	IS	N: 10 (9 receiving rtPA) Age: 67 ± 13 y Male: NR NIHSS: 5.5 [4-12].	Whole body cooling by cold saline infusion (4 °C, 25 mL/kg body weight)	Administration for 123 ± 20 min after symptom onset and 17 ± 11 min after rt-PA treatment start. Drugs: pethidine/ buspirone for preventing shivering.	NR	NR	None	None	T_Ty_ decreased from a baseline of 37.1 ± 0.7 °C by a maximum of 1.6 ± 0.3 °C (p < 0.005). The lowest measured T_Ty_ (35.4 ± 0.7 °C) was reached 52 ± 16 min after cold infusion start.	NR
**Sund-Levander and Wahren 2000** [66]	SAH, CH, TBI	N: 7 Age: 57 ± 11 y Male: 29%	Whole body cooling, or wrists, ankles, or groin cooling.	Body surface sponged with cool water or alcohol; or alcohol-saturated wraps on wrists, ankles, or groin. Additional cooling with a fan.	NICU	IRTT, Genius 3000 A, Sherwood Medical, UK	None	Skin surface at the toe tip	An increased T_Ty_ - toe T gradient was significantly associated with the occurrence of shivering (p < 0.01).	The T_Ty_ - toe T gradient decreased more during the intervention when the arms and legs were covered (9.1 ± 5.7 °C) than uncovered (11.7 ± 4.2 °C, p < 0.001).

Data are numbers (N), percentages (%), mean ± standard deviation, median (interquartile range) or [range], as available. *Hypothermia was performed for neuroprotection only in 6 patients

*CH*, cerebral haematoma; *IRTT*, infrared tympanic thermometer; *ICH*, intracerebral haemorrage; *ICP*, intracranial pressure; *IS*, ischemic stroke; *NICU*, neurointensive care unit; *NIHSS*, National Institutes of Health Stroke Scale; *NR*, not reported; *rtPA*, recombinant tissue-type plasminogen activator; *SAH*, subarachnoid hemorrhage; *SSS*, Scandinavian Stroke Scale (SSS) score; *SU*, stroke unit; *T*, temperature; *TH*, therapeutic hypothermia; *TM*, temperature measurement; *TTM*, target temperature management; *T*_*Ty*_, tympanic temperature; *TBI*, traumatic brain injury; *y*, years

### Tympanic temperature measurement during testing of TTM approaches in healthy subjects

The literature search identified 12 studies that monitored T_Ty_ to test the effects of TTM protocols in healthy subjects. These studies are summarized in Table 2. In 10 studies [32–40, 42], TTM was achieved using surface cooling garments, such as head and neck or chest and thighs cooling devices. In the remaining two studies [41, 43], endovascular cold solutions were used. Comparative core-temperature measurements were present in eight studies [32–39], where rectal/intestinal sites were monitored. Consistently among studies, T_Ty_ showed more pronounced changes than rectal [33–35] or intestinal temperature [32]. During chest and thighs surface cooling, the difference between tympanic and rectal temperature was maximal during induction of hypothermia and decreased during its maintenance [36]. Compared to other measurement sites, during head cooling T_Ty_ temperature showed lower variations than skin temperature [35] and more reliable data than sublingual temperature measurements [42]. Overall the studies showed that T_Ty_ was useful in the validation of novel cooling strategies in healthy subjects, where T_Ty_ was able to track temperature variations induced by local head and/or neck [32–35, 42] or chest and tights cooling [36–39]. In addition, it was shown to correlate with intracerebral blood flow velocity during mild hypothermia induced by local cooling [39]. In four studies focusing on TTM shivering thresholds [37, 38, 41, 43], T_Ty_ was able to identify the shivering threshold during either local [37, 38] or endovascular cooling [41, 43].

### Tympanic temperature measurement during TTM in acute cardiovascular events

Seventeen studies were identified in which T_Ty_ was measured during TTM in patients with acute cardiovascular events. The studies are summarized in Table 3. Fifteen studies [44–58] included patients with CA. TTM was started in a pre-hospital setting in four studies [45, 46, 57, 58], while it was started at the emergency department in the remaining eleven [44, 47–56]. Two studies [59, 60] included patients with ST-segment elevation myocardial infarction undergoing percutaneous coronary interventions and TTM was performed pre-reperfusion [59, 60]. In one study, the procedure was started in the pre-hospital setting [60]. In all the studies, target temperature was in the range of mild hypothermia (33–34 °C), whereas the TTM cooling procedures and protocols varied among the studies, including (i) local cooling procedures [44–46, 48, 49, 58], (ii) whole body cooling [47, 57, 59, 60], and (iii) a combination of the two [50–56]. Comparative core-temperature measurements, including esophageal, rectal, bladder, iliac, or pulmonary artery sites, were mostly available for the studies performed in hospital settings [44, 45, 47–51, 53–55], and provided indications of T_Ty_ accuracy in relation to the TTM phases [50, 51]. During local cooling procedures, such as trans-nasal cooling, the tympanic site generally displayed a faster response than the rectal and bladder ones [44, 46]. The tympanic site showed comparable cooling times with respect to the esophageal site [49], although it showed larger temperature variations in response to the cooling maneuvers [44, 49]. T_Ty_ showed larger bias compared to esophageal temperature during head and especially head-neck cooling, where it underestimated esophageal T with an average bias of −1.65 °C and −3.06 °C (p=0.001), respectively [54]. During whole body cooling, the tympanic site showed a low average bias (0.021 °C) and high correlation (r = 0.95, p < 0.0001) compared to the esophageal site [47]. Conversely, T_Ty_ showed the highest bias in comparison with pulmonary-artery measurements [50, 51], resulting in the underestimation of core temperature through the different TTM phases (overall bias of − 0.6 °C [50] and − 1.03 °C [51]) and in a shorter cooling-time duration [51]. In pre-hospital settings, T_Ty_ was capable of tracking the effects of prompt post-ROSC application of TTM by cold saline infusion [57] or by a hypothermia cap [58] in CA patients, as well as the effects of cold saline and surface pads in patients with acute myocardial infarction [60]. However, tympanic measurements showed to be biased by external factors, such as variations in the environmental temperature [52] or the presence of snow/ice in the ear canal [45]. In the in-hospital setting, tympanic measurements were able to track temperature changes associated with nasal/pharyngeal or head/neck cooling [44, 48, 49, 53], cold saline infusion [47, 57], or a combination of local and whole body cooling [50, 51, 53–56]. In patients with acute myocardial infarction [59, 60], the tympanic site was used to complement catheter tip measurements, when the latter were not available.

### Tympanic temperature measurement during TTM in acute neurological disorders

Six studies tracked T_Ty_ during TTM in patients with acute neurological disorders, which included ischemic or hemorrhagic stroke, subarachnoid hemorrhage, and cerebral hematoma after traumatic brain injury. The studies are summarized in Table 4. TTM protocols were applied in-hospital in all the retrieved studies. In three studies [61–63], patients were intubated and deeply sedated. TTM protocols differed among the studies in terms of cooling devices, target temperature measurement sites, starting temperature, and target temperature (mostly mild hypothermia). The applied cooling techniques included (i) whole body cooling by intravenous injection of cold saline solutions [62, 65] and (ii) local body cooling by nasopharyngeal [61, 62] or head/neck cooling devices [63], body surface wraps/sponges [66], and/or the “forced air” method [64]. In most of the studies, comparative core measurements were available for the bladder, rectal, and esophageal sites [61–63, 67], and in one study also for the pulmonary artery [61]. T_Ty_ showed a larger drop compared to other core-temperature measurement sites during pharyngeal cooling [61], while it showed strong correlation with rectal temperature during surface cooling by “forced air” method [64]. In three studies [61–63], brain temperature measurements from a probe inserted below the cortical surface were available. T_Ty_ correlated well with brain temperature during whole body cooling induced by intravenous cold saline solution in stroke patients, although it displayed lower values already at baseline with a bias of − 0.4 °C [62]. During nasopharyngeal cooling [61, 62] or head and neck cooling [63], T_Ty_ overestimated brain cooling, showing a more marked decrease (drop in the first hour of cooling ranging from −1.69 to −2.2 °C at the tympanum vs. −0.32 to −1.4 °C at the brain), while other core-temperature measurement sites underestimated brain cooling, displaying a lower decrease (temperature drop ranging from − 0.18 °C to − 1.1 °C) [61, 63].

T_Ty_ displayed capability to track temperature changes induced by either local or whole cooling. In addition, when used in combination with skin temperature, it depicted the risk of shivering during surface cooling [66].

## Discussion

The main findings of the present scoping review, aimed at assessing the diffusion, feasibility, and accuracy of T_Ty_ monitoring during TTM, are: (i) T_Ty_ was capable to track temperature changes induced by a variety of TTM approaches, including local or whole body cooling, in both pre-hospital and in-hospital settings and under different clinical conditions; (ii) T_Ty_ may have selective advantages for TTM in pre-hospital settings, where it is often the sole temperature measurement available; and (iii) limited evidence is available about T_Ty_ accuracy in relation to reliable core body and brain sites.

### Feasibility and performance of T_Ty_ monitoring in emergency and critical care

The evidence provided by the 35 identified studies generally supported the capability of T_Ty_ to follow temperature changes induced by either local or whole-body cooling strategies. The most common application fields for T_Ty_ were the testing of novel cooling strategies in healthy subjects and the monitoring of TTM in patients with acute cardiovascular events, while applications in patients with acute neurological disorders were sparser. In patients with acute cardiac disease, T_Ty_ monitoring was applied in both pre-hospital and in-hospital emergency settings. In the former setting, tympanic monitoring was mostly used as the sole temperature measurement, which may indicate a selective advantage of T_Ty_ in this condition. Thanks to its reduced invasiveness and easy application, ear probe measurements may allow prompt TTM initiation [68]. In comparison, esophageal temperature probes usually require an intubated patient and rectal temperature measurements may be not easily accessible [68]. However, factors limiting T_Ty_ reliability should be properly considered for an appropriate use of the technique. T_Ty_ measurements may be influenced by alterations in the blood flow to the brain, as demonstrated for instance by tilting maneuvers [23]. Therefore, T_Ty_ measurements in CA patients should be considered reliable only after the patient has regained a stable spontaneous circulation. Moreover, pre-hospital studies showed T_Ty_ measurements to be affected by external factors, such as variations in the environmental temperature or the presence of snow/ice in the ear canal [45]. Consistently, previous studies pointed out the necessity of performing T_Ty_ measurements in a clean and dry ear canal and the importance of properly insulating the tympanic probe, especially when operating in settings exposed to environmental factors (e.g., cold, wind) [25, 69].

### Comparison of the tympanic site versus other core-temperature measurement sites

In emergency and critical care settings, alternative core-temperature measurement sites are available to track temperature; thus, the performance and eventual advantages of T_Ty_ in comparison to other measures need to be evaluated. Tympanic measurements were combined with other measurements in 30 studies [32–39, 41–55, 59–64, 66], of which 22 studies [32–39, 44, 46–54, 61–64] provided a direct comparison with temperatures measured at different core or brain sites. These studies presented heterogeneity in terms of studied population, cooling protocols and devices, tympanic thermometer type (IRTTs or thermistor/thermocouple thermometers), and comparative/reference sites. All these variability factors hindered the calculation of an overall figure of merit for tympanic measurement site. As an additional limitation, rectal temperature was mostly used as comparator among studies. The rectal site has known limitations and a slow response in dynamic conditions [20, 70–72], which is mainly attributed to the buffering influence and heat-sink effect of rectal tissue and stool in the rectum [73, 74].

The comparison of T_ty_ with the most reliable core sites (i.e., esophageal, jugular, and pulmonary artery sites), available from studies in emergency and critical care settings, showed that T_Ty_ performance depended on the cooling protocols and reference site considered. Compared to esophageal temperature, T_Ty_ showed comparable cooling times during intranasal cooling [49], and low bias and high correlation during cold saline infusion [47]. A larger bias with esophageal temperature was observed during head and neck cooling [54]. Compared to the pulmonary artery, the tympanic site showed the highest bias during cold saline infusion [50, 51]. Although the pulmonary artery temperature is usually considered as the gold standard core-temperature to guide clinical mild hypothermia [75, 76], previous studies have shown that the temperature of the pulmonary artery blood may reflect less well brain temperature when hypothermia is induced or reversed, during either selective head cooling or rapid intravascular cooling [77, 78]. Instead, esophageal temperature responds rapidly to changes in the temperature of the blood perfusing the heart and great vessels [77, 79] and it showed a better relationship with brain temperature when inducing hypothermia and at early TTM, in either selective head or whole-body cooling [77]. The better agreement of T_Ty_ with the esophageal than with the pulmonary artery temperature may thus suggest the reliability of T_Ty_ to track brain temperature. The capability of T_Ty_ to reflect brain temperature is supported by the assumption that the tympanic membrane is supplied by vasculature from the same sources that supply the brain (i.e., branches of the basilar and internal carotid arteries, which join anastomoses with branches of the external carotid artery in the region around and within the tympanic membrane [21, 22, 80]), which guarantees thermal equilibrium between the two sites [23]. However, although T_Ty_ is currently the most commonly used non-invasive method for brain temperature estimation [81], being the sole anatomical structure close to the brain that is accessible without surgery [82, 83], concerns remain on its accuracy, mainly due to measurement errors, measuring devices, and/or real temperature differences between the ears [80]. In the specific setting of TTM, the present review revealed a gap of evidence in the literature about the capability of T_Ty_ to reflect brain temperature. We identified only three studies in acute neurological patients [61–63], which provided comparative direct brain temperature measurements at a single subcortex site. The studies displayed heterogeneity in terms of patient characteristics and underlying disorders, cooling procedures, and target temperature. The results showed a high correlation between T_Ty_ and brain temperature during whole body cooling. Nonetheless, T_Ty_ generally overestimated brain cooling in either whole body [61, 62] or local body cooling [62], with more severe overestimation during head and neck cooling [63]. Of note, other sites for core-temperature measurements generally underestimated cooling effects [61]. Although these results may suggest the potential of T_Ty_ to track brain temperature with similar performance to other more invasive distal measurement sites, the larger response of T_Ty_ may result in an overestimation of cooling effects through different TTM phases and thus in a shorter cooling-time duration [51], with the risk for patients to stay outside the ideal temperature range during TTM induction and steady state.

### Future perspectives

Simultaneous measurements at different brain and core temperature sites according to well-defined protocols should be performed during both local and whole-body cooling procedures. The characterization of the spatiotemporal temperature patterns under various TTM approaches by a continuous temperature acquisition through the different TTM phases is desirable. In experimental studies, brain temperature should be monitored at multiple sites, since a single site may not reflect temperature across the brain, especially in the presence of head cooling and marked temperature gradients [84–86]. The systematic assessment of bias and correlation between T_Ty_ and brain or other core-temperature measurement sites and the comparison with therapeutic outcome may allow to define sharp recommendation and safe target ranges for T_Ty_ under different TTM applications. T_Ty_ performance may be improved with proper recalibration of target temperature values, as T_Ty_ often led to an underestimation of core temperature even at baseline but showed a moderate to high correlation with esophageal temperature. Finally, clinical, experimental, and industrial research should synergistically concur to develop wearable temperature trackers [80], able to overcome the limitations of current tympanic thermometers [25, 66, 69, 80, 87] and to grant fix probe positioning and protection from external environmental conditions [80]. These developments may improve temperature monitoring and allow early TTM extension under logistically challenging critical conditions.

## Conclusions

The results of the present scoping review provided evidence about the capability of T_Ty_ to track temperature changes induced by either local or whole-body cooling in both pre-hospital and in-hospital TTM applications. However, there is a paucity of studies performing a systematic comparison of T_Ty_ performance with reliable core and brain temperature measurement sites, which hinders a thorough evaluation of T_Ty_ advantages in emergency settings and of the capability of T_Ty_ to track brain temperature. Future experimental and clinical studies should bridge this gap of evidence by providing reliable devices and dedicated temperature ranges for safe application of T_Ty_ in TTM and by clarifying the relationship between T_Ty_ and brain temperature. Thanks to its easy use and reduced invasiveness, T_Ty_ may have selective advantage in pre-hospital settings, when practical limitations may hinder temperature acquisition from more invasive sites.

## Data Availability

All the data generated or analyzed during this study are included in the published article.

## Abbreviations

ATP: Adenosine triphosphate
CA: Cardiac arrest
IRTT: Infrared tympanic thermometer
PCC: Population-Concept-Context
ROSC: Return of spontaneous circulation
TTM: Target temperature management
T_Ty_: Tympanic temperature
